# Feasibility of an online mindfulness-based program for patients with melanoma: study protocol for a randomised controlled trial

**DOI:** 10.1186/s13063-018-2575-x

**Published:** 2018-04-13

**Authors:** Lahiru Russell, Anna Ugalde, Donna Milne, Meinir Krishnasamy, Eric O (Seung Chul), David W Austin, Richard Chambers, Liliana Orellana, Patricia M Livingston

**Affiliations:** 1 Deakin University, School of Nursing and Midwifery, Faculty of Health, Geelong, Australia; 2 Skin and Melanoma Services/Department of Cancer Experiences Research, Peter MacCallum Cancer Centre, Melbourne, Australia; 3 Department of Nursing, School of Health Sciences, University of Melbourne, Melbourne, Australia; 4 Deakin University, School of Psychology, Faculty of Health, Geelong, Australia; 5 Campus Community Division, Monash University, Clayton, Australia; 6 Deakin University, Biostatistics Unit, Faculty of Health, Geelong, Australia; 7 Victorian Comprehensive Cancer Centre, Melbourne, Victoria Australia

**Keywords:** Mindfulness, Internet-based, Feasibility, Pilot, Cancer, Melanoma

## Abstract

**Background:**

People with a melanoma diagnosis are at risk of recurrence, developing a new primary or experiencing disease progression. Previous studies have suggested that fear of a cancer recurrence is clinically relevant in this group of patients and, if not addressed, can lead to distress. Mindfulness-based interventions have been shown to alleviate symptoms of anxiety and depression among various groups of cancer patients. Online mindfulness-based interventions have the potential to reach people unable to attend face-to-face interventions due to limitations such as cancer-related illness, transportation or time constraints. This study aims to (1) examine whether individuals with a melanoma diagnosis are willing to participate and adhere to a 6-week online mindfulness-based intervention and (2) explore potential benefits of the program on fear of cancer recurrence, worries, rumination, perceived stress and trait mindfulness to inform the design of a clinical trial.

**Methods/design:**

This is a single-site randomised controlled trial of a feasibility study. Seventy-five participants with stage 2c or 3 melanoma will be recruited from a melanoma outpatient clinic and randomised (2:1) either to an online mindfulness-based program (intervention) or to usual care (control). The intervention is a 6-week program specifically developed for this study. It consists of videos describing the concept of mindfulness, short daily guided meditation practices (5–10 min), automated meditation reminders and instructions for applying mindfulness in daily life to enhance wellbeing. All participants will complete questionnaires at baseline and at 6-week post-randomisation. Participants in the control group will be given access to the online program at the end of the study. Primary outcomes are overall recruitment; retention; extent of questionnaire completion; and usability and acceptability of, and adherence to, the program. The secondary outcomes are fear of cancer recurrence, worries, rumination, perceived stress and trait mindfulness measured using validated instruments.

**Discussion:**

This feasibility study will evaluate participants’ satisfaction with the program and identify barriers to recruitment and adherence. The recruitment and data collection process will highlight methodological aspects to address in the planning of a larger scale study assessing the impact of an online mindfulness-based intervention on fear of cancer recurrence and wellbeing.

**Trial registration:**

Australian New Zealand Clinical Trials Registry, ACTRN12617000081314. Registered on 16 January 2017.

**Electronic supplementary material:**

The online version of this article (10.1186/s13063-018-2575-x) contains supplementary material, which is available to authorized users.

## Background

Melanoma affects over 232,000 people worldwide each year [1]. People who have received treatment for melanoma are up to nine times more likely to develop a new melanoma, and this risk remains elevated for more than 20 years after the initial diagnosis [2]. The risk that the melanoma comes back locally or in another part of the body after it has been treated (i.e. recurrence) is related to the clinical characteristics of the tumour (i.e. thickness, ulceration and spread) and is estimated overall to be 9% [2]. Thick primaries, ulcerated lesions or regional lymph node metastases, corresponding to stages 2b, 2c, 3 or 4 according to the melanoma TNM staging system [3], have a high risk of relapse with 5-year mortality rates of 20–80% [3].

Fears and concerns about a melanoma recurrence are common among melanoma survivors. In a large multicentre study examining the supportive care needs, anxiety, depression and quality of life among patients diagnosed with melanoma, the main concerns were the risk of recurrence (17%), fear of metastatic disease (17%) and uncertainty regarding outcomes if metastatic disease is detected (16%) [4]. Furthermore, a high proportion (72%) of melanoma survivors with a history of at least one diagnosis of melanoma reported clinically relevant fear of cancer recurrence (FCR) [5].

FCR is a rational and normal response to a stressful event, but if the fear persists and is not resolved through coping or adaptation, it may lead to psychological distress such as anxiety or depression [6]. In patients with melanoma, psychological distress is associated with delay in seeking medical advice [7] and poor psychological adjustment, which is related to disease progression through immunological response to stress [8].

FCR is often viewed as a multidimensional phenomenon, with an emotional component of anxiety and cognitive aspects of worry, preoccupation and intrusive thoughts [9]. Maladaptive information processing styles, such as worrying or ruminating uncontrollably over cancer-related information, were positively correlated with FCR [10]. A theoretical formulation of FCR, proposed by Fardell and colleagues, suggests that attentional bias towards worrisome and unhelpful thoughts underlies the association between maladaptive information processing and FCR [11]. According to this formulation, teaching skills to cultivate attentional flexibility and mindful awareness of thoughts could have therapeutic benefits [11]. These skills are at the core of mindfulness-based interventions [12].

Mindfulness is a state of mind in which one pays attention to the present moment in a non-judgmental, curious, accepting and decentred way [12–14]. In this state, thoughts and feelings are observed as temporary events in the mind, instead of a reflection of oneself or reality. Through detached self-observation, individuals learn to reflect on situations and are able to respond in more adaptive ways instead of reacting in an automatic, habitual pattern [12]. Hence, the practice of observing one’s thoughts, emotions and body sensations, with discernment rather than judgment, is believed to be a starting point linking mindfulness practice and psychological wellbeing [15, 16]. Supporting this idea, a meta-analysis exploring the mechanisms underlying the therapeutic effects of mindfulness-based interventions (MBIs) on psychological outcomes demonstrated that mindfulness positively mediates the effects of the interventions on mental health outcomes, such as depression, stress and anxiety, while rumination and worry negatively mediate these same outcomes [17].

In MBIs, the cultivation of mindfulness is practised in two distinct and concurrent ways, namely formal and informal practices. The formal practice of mindfulness meditation can be done in different ways (e.g. walking, movement, sitting) [12, 18, 19]. These practices strengthen attention regulation and allow observation of mental habits such as rumination [18, 19]. As the practice evolves, the meditator gains clarity of mind and greater awareness of his or her mental and emotional fluctuations, enabling them to alter reactive cognitive and emotional habits related to mental distress [20].

Through informal practice, the individual is encouraged to bring a similar approach of awareness to daily activities. Paying attention to the sensory experience of each moment keeps the attention more in the present moment and reduces worry and rumination [12]. Regular meditation builds the attention foundation for informal practice, and together they lead to increased mindfulness in everyday life [21, 22].

In oncology settings, a meta-analysis of 22 studies on patients with different cancer types and stages reported moderate benefits of MBIs in reducing symptoms of anxiety and depression [23]. A recent trial demonstrated the efficacy of a MBI in assisting breast cancer survivors to cope with their fear of recurrence [24], with participants randomly assigned to a 6-week MBI showing significant improvements in FCR.

Besides the growing body of evidence on the benefits of MBI for individuals living with cancer, practical barriers exist that may diminish access to, and participation in, face-to-face programs. Generally, online interventions are more easily accessible, are available at any time to people in their own environment, and enable people to work at their own pace and to remain anonymous. In addition, online MBIs are less costly because they do not necessarily require the involvement of a trained therapist educated in mindfulness [25–27]. In a cross-sectional survey of 291 individuals attending a melanoma clinic, 44% of the participants reported interest in participating in an online meditation-based program, indicating that this type of intervention might be acceptable in a similar cohort [28]. An online Mindfulness-Based Cancer Recovery program initially tested for feasibility with distressed participants completing treatment for different types of cancer showed positive effects on mood disturbance, stress symptoms, spirituality and mindfully acting with awareness [29].

The current study will assess the feasibility of delivering an online mindfulness-based program to patients affected by melanoma. It will examine patterns of usage and content relevance to inform the design of a larger randomised control trial.

### Aims and objectives

The overall aim of the study is to examine the feasibility and acceptability of an online mindfulness-based program among people who have completed treatment for melanoma.

The primary objective of the study will be to assess the feasibility of conducting a larger scale randomised control trial by evaluating:

- Overall recruitment, retention and questionnaire completion rates
- Adherence to, and usability and acceptability of, the online program

The secondary objectives will be to assess the potential benefit of the intervention on fear of cancer recurrence, worry, rumination, perceived stress and trait mindfulness compared to a usual-care group and to provide effect size and variance estimates for these outcomes to inform the design of a larger clinical trial.

## Methods/design

### Design

The development of the study protocol followed the SPIRIT (Standard Protocol Items: Recommendations for Interventional Trials) guidelines [30] (Additional file 1). The description of the intervention followed the TIDieR (Template for Intervention Description and Replication) guidelines [31] (Additional file 2).

**Fig. 1:**
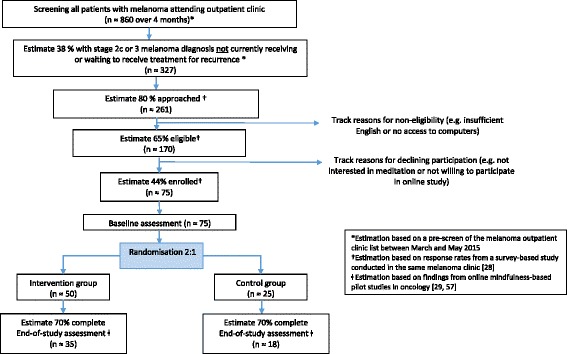
Participant flow diagram and recruitment estimates

This study is a single-centre, randomised controlled pilot study. The control group will continue to receive care from their treatment centre as usual, while participants in the intervention group will receive access to a 6-week online mindfulness program as well as their usual care. The groups will be compared to assess the potential effect of the intervention on a series of outcome measures. Measurements will take place at baseline (i.e. before randomisation) and at the end of the study (i.e. 6 weeks after randomisation). In the intervention group, participant engagement with, and usability and acceptability of, the program will be assessed weekly through a short questionnaire. The overall design and participant flow through the trial is illustrated in Fig. 1.

Clearance to conduct this study was granted by the human research ethics committees of the recruitment centre (HREC/16/PMCC/139) on 4 January 2017 and by Deakin University (DUHREC 2017-036) on 6 February 2017.

### Eligibility criteria

Participants will be included if they have a melanoma diagnosis of stage 2c, 3a, 3b or 3c and completed their last treatment within the past 5 years, are 18 years or older, have sufficient understanding of English to consent and participate, and have access to the Internet.

Patients with a history of distant metastasis will be excluded as the disease trajectory of stage 4 melanoma remains unpredictable and generally incurable when compared to other stages with high risk of recurrence [32, 33]. Patients will also be excluded if they have a severe cognitive impairment or intellectual disability, as reported in the medical record or determined by the treating clinician or oncology nurse, or are due to commence or currently receiving treatment for a melanoma recurrence or a second melanoma.

### Setting and recruitment process

Recruitment will take place in the melanoma outpatient clinics of a major metropolitan cancer centre in Melbourne, Australia. Patients will be identified through the melanoma outpatient database and study eligibility determined using electronic medical records.

During the follow-up consultation, clinicians will inform patients of their eligibility and will invite them to meet with the researcher at the end of the consultation to learn more about the study.

Patients will be reminded that participation is voluntary and their decision to participate or not will have no impact on their clinical care. A patient information package will be provided to each interested patient, and the researcher will follow up by telephone as agreed with patients to answer any questions related to the project.

Upon providing online consent to participate, a unique study identification number will be automatically generated for each participant. This identification number will be generated by a SSL-certified server to ensure privacy and integrity of the data and will not contain participants’ personal information. Participants will then be directed to the baseline questionnaire and upon completion will be randomised into either the intervention or the control group. Participants will also receive an email confirming the condition they are assigned to. The email for the intervention group will also comprise a URL address including the unique study identification number to access the intervention.

### Randomisation

Participants are randomised (2:1) to the intervention or control group stratified by sex, time since treatment completion (≤12 months, >12 months), and meditation experience (yes/no). A random sequence will be generated for each one of the eight strata using block randomisation. For every three participants enrolled in each stratum, two will be allocated to the intervention group and one to the control group. Random sequences will be embedded in the online system, ensuring allocation concealment. Study personnel will not have access to the sequences and will be blind to group assignment. After randomisation, participants will be unblinded to group assignment, as the intervention does not allow for blinding. All questionnaires will be completed online, which will reduce the influence of researcher bias. Given the nature of the intervention and the context in which participants are recruited, i.e. outpatient clinic, we cannot rule out the usual risks of bias due to participants not being blinded to treatment allocation. However, in this study, all surveys will be completed online; health practitioners have no involvement in the collection or assessment of any of the trial outcomes. Unequal randomisation, where the number of participants allocated to the intervention group exceeds that in the control group, has been identified as a suitable approach in early-phase studies where the aim is to explore various dimensions of the intervention, such as patterns and frequencies of usage [34].

### Intervention group—online mindfulness-based program

The intervention was developed for the purpose of this study by the authors, including a clinical psychologist (RC) with expertise in delivering mindfulness-based programs (e.g. [35]), cancer nurses and researchers (AU, LO, DM, MK and PL) with expertise in developing and evaluating cancer supportive care interventions (e.g. [36–38]), and a psychology researcher (DA) and website developer (EO) with expertise in developing and evaluating online interventions (e.g. [39]). The website content was initially developed by two of the authors (RC and LR) and finalised by consensus.

The intervention is a 6-week online mindfulness-based program designed to (1) help patients understand the potential benefits of using mindfulness in their day-to-day life and (2) support the establishment of daily meditation practice. Each week of the program explores a different topic, which builds on the topics explored in former weeks.

The program includes three weekly components: (1) an *educational* component to increase participants’ knowledge about the meaning of mindfulness, its application in everyday life and potential benefits; (2) a *formal meditation practice* to strengthen attention regulation and learn to observe mental habits such as rumination or worry; (3) an *informal practice* to encourage participants to integrate mindfulness into their daily activities. Table 1 describes these components in further detail.

**Table 1:** Mindfulness program weekly schedule

	Educational components	Weekly video topics (length of video in seconds)	Formal practice—twice daily meditations	Informal practice—practice during daily activities
Week 1	Introduction to mindfulness	• Welcome (27) • What is mindfulness (105) • How can mindfulness help (53) • Example of a meditation practice (141) • Debriefing the meditation practice (55) • Practice of the week (25)	Body scan (5 min)	Practise being present, noticing moments of being mindful
Week 2	Reducing stress with mindfulness	• Reflecting on last week (28) • The stress response (96) • What happened to the body during the stress response (89) • How can mindfulness help (50) • Practice of the week (88)	Breath and body (5 min)	Noticing when feeling stressed, how the body is responding, what sort of thought activated the stress
Week 3	Relating mindfully to emotions	• Emotions (28) • Mindful ways to regulate your emotions (193) • Practice of the week (28)	Working mindfully with emotions (10 min)	Noticing when caught up in emotions and practising coming back to the present moment
Week 4	Self-compassion	• Self-criticism (126) • Self-compassion (68) • Where does mindfulness come in (34) • Practice of the week (44)	Body, breath and sound (10 min)	Noticing when involved in self-criticism and practise using self-compassion
Week 5	Communicating mindfully	• Communication (93) • Practice of the week (51)	Body, breath, sound and thoughts (10 min)	During conversation, notice when the mind is caught up with other thoughts. Practise bringing attention back to the conversation
Week 6	Living mindfully	• The mindful pause (40) • Everyday mindfulness (52) • Meditation practice (29) • An ongoing experience (62)	Tuning into your surroundings (10 min)	Pause throughout the day (1-min practice). Get into the habit of watching the contents of your mind

Participants will receive an automatically generated email at the end of each week with a link to a brief questionnaire inquiring about their level of engagement with the program. Following completion of the questionnaire, participants will be directed to the website. Each topic of the program is explained through a short video ranging from 25 s to 3 min. The script of the videos is available for downloading and printing in PDF format so that participants can keep a copy of it should they wish to use it at a later stage. At the end of each video, participants will be reminded to continue with their daily meditation practice (formal practice) and will be given a specific mindfulness task to practise during their daily activities (informal practice).

To support participants with establishing a meditation routine, emails containing a link to a short, guided meditation audio file, voiced by co-author RC, will be sent twice daily. These emails will serve as reminders to meditate and will provide easy access to the meditation practice of the week.

The website has a section addressing common questions about meditation, which is available at any time throughout the course of the program. The development of this section was informed by (1) answers to a survey conducted in a similar study population about the practice of meditation [28] and (2) questions raised by participants of a commercially available meditation-based program designed for new meditators in the general population [40].

Participants will have access to the mindfulness program at any time every day of the week via the hyperlink sent in the emails.

### Control group—usual care

Participants in the control group will continue to receive their usual care. They will complete baseline and post-6-week assessments. Upon completion of the final assessment, the control group will be offered the online mindfulness program.

### Study discontinuation

Reasons for which participants may wish to discontinue the program may be varied, e.g. lack of time, lost interest in the program, or feeling too unwell. Participants who wish to discontinue will be invited to voluntarily inform the study team by email and provide reasons for discontinuation. Due to the self-guided, anonymous and automated nature of the intervention, the study team will not be able to initiate communication with participants who ceased interacting with the website.

### Data collection

**Fig. 2:**
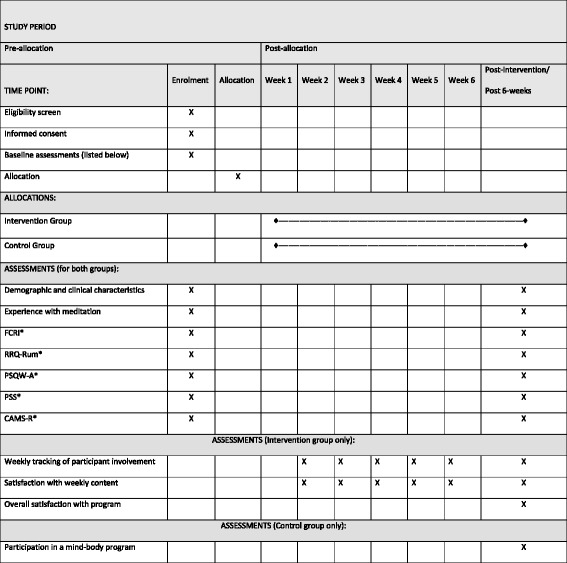
Overall schedule and time commitment for trial participants

Figure 2 illustrates the overall schedule and time commitment for trial participants in both groups.

All assessments will be performed online through the Qualtrics platform. At baseline, participants’ demographic information (i.e. sex, age, education, marital status and current employment status), previous meditation experience and preferred time to receive email reminders will be obtained. Clinical information (i.e. stage of melanoma, dates of diagnosis and treatment, types of treatment received and time since the end of treatment) will be collected from medical records, with participants’ consent.

In order to retain patients’ anonymity, all data will be non-identifiable and stored in password-protected, encrypted files at Deakin University.

### Outcome measures

#### Fear of Cancer Recurrence Inventory

This 42-item Fear of Cancer Recurrence Inventory (FCRI) questionnaire is a multidimensional FCR scale intended for use with all cancer patients. Items were developed on the basis of a cognitive–behavioural formulation of FCR [41]. The FCRI consists of seven domains: triggers, severity, psychological distress, functional impairment, reassurance, insight and coping strategies. It has shown high internal consistency and good construct and criterion validity in a group of adults with different cancer types [42] and in adults with a history of melanoma [5].

#### Rumination/Reflection Questionnaire – Rumination subscale

Ruminative thoughts are measured using the 12-item Rumination subscale that measures “ruminative self-attention”, the tendency to dwell on, rehash or re-evaluate events or experiences [43]. Items are rated on a 5-point Likert scale from “strongly disagree” (1) to “strongly agree” (5). The scale showed good internal reliability in a sample of newly diagnosed colon cancer patients and mixed cancer patients (Cronbach’s *α* = 0.90 and 0.92, respectively) [44, 45]. In a sample of women post-cancer treatment, where a majority were breast cancer patients, Rumination/Reflection Questionnaire – Rumination subscale (RRQ-Rum) scores were negatively correlated with mindfulness change scores (*r* = −0.34, *P* = 0.03) [46].

#### Penn State Worry Questionnaire – Abbreviated

Worries are measured using the Penn State Worry Questionnaire – Abbreviated (PSWQ-A), an eight-item scale [47] adapted from the PSWQ that assesses trait worry by capturing the excessiveness, prevalence and uncontrollability of clinical worry [48]. Items on the PSWQ-A are rated from 1 (not at all) to 5 (very typical), and total scores range from 8 to 40, with higher scores indicating greater worry. The PSWQ-A is a valid and reliable measure for use among older adults [47] and women with cancer [49] with internal consistency of 0.87 and 0.96, respectively.

#### Cognitive and Affective Mindfulness Scale – Revised

Trait mindfulness is measured using the Cognitive and Affective Mindfulness Scale – Revised (CAMS-R), a 10-item self-report questionnaire. This scale uses everyday language appropriate for those with little meditation experience and is designed to capture mindfulness as a general daily experience [50]. The questionnaire comprises four domains of mindfulness (attention, present-focus, awareness and acceptance/non-judgment). Participants are asked to rate on a 4-point Likert scale how much they relate to each statement. Possible responses are 1 = rarely/not at all, 2 = sometimes, 3 = often and 4 = almost always.

Compared to other measures of mindfulness, the CAMS-R is unique in that it is related to psychological distress [51], which is highly relevant to the current study population [52].

#### Perceived Stress Scale

The Perceived Stress Scale (PSS; 10-item version) is a self-report questionnaire measuring the global perception of stress [53]. The items were designed to evaluate how unpredictable, uncontrollable and overloaded respondents find their lives. The scale has internal consistency ranging from 0.75 to 0.86 [54]. Consistent with this range, studies measuring perception of stress in people with prostate cancer [55] and breast cancer [56] had Cronbach’s *α* of 0.78 and 0.87, respectively.

#### Assessment of intervention content relevance

At the end of each week, a short questionnaire will ask participants if the weekly topic was beneficial and what aspects of the program were liked or disliked. At week 6, additional three questions ask about the overall helpfulness of the mindfulness program and feature that were the most liked or disliked.

#### Adherence tracking and meditation log

Google Analytics will be used to track participants’ online activity, including login date/times, navigation patterns, page views and duration, and features used (video, audio and document downloads). At the end of each week, a set of three questions will capture information on any use of other types of meditation unrelated to the intervention, and frequency and duration of practice. Another three questions will estimate the amount of informal practice over the past week, and the specific use of the weekly topic, using a 5-point Likert scale.

#### Controlling for existing mindfulness experience in the control group

In order to control for the potential influence of an external mindfulness-based program in the control group, participants will answer a question included in the end-of-study questionnaire about whether they have enrolled in a mindfulness-based program during the past 6 weeks.

### Sample size

We aim to enrol 75 participants over a 5-month period. The target sample size of 75 participants (50 in the intervention group, 25 in the control group) meets recommendations for pilot studies aimed at evaluating feasibility and estimating variance to inform sample size and power calculations for a main trial [57]. The 5-month recruitment period was informed by (1) screening past melanoma clinic lists for eligible patients at the cancer centre over a period of 3 months, (2) findings from a survey-based study among people with melanoma [28], and (3) an estimated 70% participation rate based on findings from online mindfulness-based pilot studies for patients with cancer [29, 58] (Fig. 1).

### Analysis plan

Descriptive statistics will be used to present participants’ characteristics. Demographic and clinical characteristics, and experience with meditation will be compared between intervention and control groups, using *t* test or Wilcoxon rank-test (for continuous variables) depending on the outcome distribution and chi-square or Fisher’s exact test (for categorical variables).

#### Primary objective—feasibility

Descriptive statistics will be used to present feasibility data. The overall study feasibility will be assessed through the following measures:

- Proportion of people approached (target: 80%): number of people approached as a proportion of those meeting clinical eligibility. Reasons for not approaching will be summarised.
- Proportion of people eligible for the study (target: 65%): number of people meeting all eligibility criteria as a proportion of those approached. Reasons for non-eligibility criteria and declining participation will be summarised.
- Proportion of people enrolled (target: 45%): number of people completing the baseline questionnaire as a proportion of those eligible.
- Completion rate (target: 70%): number of participants completing the end-of-study assessment as a proportion of those enrolled. It will not be possible to capture reasons for non-completion as there will be no interaction between the researcher and participants once they are enrolled in the study.

The intervention feasibility will be assessed through the following measures:

- Meditation adherence rate (target: 70%): number of guided meditations listened to per week over the total number of meditation sessions scheduled (14 sessions/week)
- Rate of adherence to weekly sessions (target: 50%): the number of videos viewed over the total number of videos scheduled per week
- Engagement with the intervention: recording website navigation patterns and features used (video, audio and document downloads)
- Content relevance: type of benefits experienced and aspects of the program most liked and disliked (data collected through weekly questionnaires)

#### Secondary objectives—potential efficacy of intervention on outcome measures

To explore the potential efficacy of the intervention on any of the secondary outcomes (i.e. fear of cancer recurrence, worry, rumination, perceived stress and trait mindfulness), a linear mixed model will be fitted including group (intervention, control), time (pre, post) and the interaction group by time as fixed effects and participant as a random effect. We will additionally adjust for any characteristics or scores showing clear evidence of imbalance at baseline. All analyses will be undertaken on an intention-to-treat (ITT) basis, i.e. participants will be analysed as randomised regardless of adherence.

In addition, potential association between overall meditation time and the secondary outcome measures will be assessed in the intervention group. Weekly meditation time will be defined as the maximum between time collected from Google Analytics and time self-reported in the weekly questionnaires.

Results of this study will be prepared in accordance with the CONSORT 2010 statement: extension to randomised pilot and feasibility trials [59].

### Data monitoring

Given the short length of the intervention and the length of the data collection period, an external data monitoring committee is not considered necessary and an interim analysis will not be performed. Nonetheless, members of the research team are regularly updated with recruitment status and study progression.

### Managing potential risk associated with participation

In the event that a participant becomes upset or distressed as a result of his/her participation, he/she can contact the researcher by email or phone for assistance. Counselling or other appropriate support will then be provided by the clinic staff who are not members of the research team. In addition, participants will receive in the study information pack contact for external support services such as Lifeline services or mindhealthconnect.org.au.

### Protocol amendments

Potential protocol modifications will be submitted for approval by the relevant human research ethics committees before being implemented.

### Dissemination policy

The research team intend to disseminate outcomes from this study in peer-reviewed journals and at relevant conferences. Participants will have access to study results on request.

## Discussion

This is the first study to address the specific issue of FCR among people with melanoma using an online MBI. In the emerging field of online MBIs in oncology, many fundamental questions specific to the online delivery mode remain unanswered, including (1) which aspects of an online MBI are the most engaging and (2) can patients learn the basic skills of mindfulness and benefit from a mindfulness program without interacting with a trained therapist? This study aims to answer these questions.

This paper describes the protocol for a pilot RCT with embedded process evaluations aiming to assess eligibility, enrolment and retention rates, barriers to recruitment, questionnaire completion rate, and intervention participation and content relevance. This study will support the design of a full-scale RCT with a longer follow-up period, and information collected from the recruitment process will highlight methodological aspects to address in the planning of a larger scale study. Participants’ interaction with the website and engagement with the intervention will inform whether this self-managed program has the capacity to engage participants into learning about and practising mindfulness skills. This information is collected on a weekly basis and will allow to address specific aspects of the program.

Given the positive impact of face-to-face MBIs on cancer patients, an online approach has the potential to provide similar support to individuals unable to attend face-to-face programs.

### Trial status

Recruitment commenced in February 2017 and is ongoing.

## Additional files

Additional file 1: SPIRIT 2013 Checklist. (DOC 121 kb)

Additional file 2: The TIDieR (Template for Intervention Description and Replication) Checklist. (DOCX 31 kb)

## Abbreviations

CAMS-R: Cognitive and Affective Mindfulness Scale – Revised
FCR: Fear of cancer recurrence
FCRI: Fear of Cancer Recurrence Inventory
MBI: Mindfulness-based intervention
PSQW-A: Penn State Worry Questionnaire – Abbreviated
PSS: Perceived Stress Scale
RRQ-Rum: Rumination/Reflection Questionnaire – Rumination subscale
